# Properties of Conductive Polyacrylonitrile Fibers Prepared by Using Benzoxazine Modified Carbon Black

**DOI:** 10.3390/polym12010179

**Published:** 2020-01-09

**Authors:** Damiro Ahn, Hyun-Jung Choi, Ho-dong Kim, Sang Young Yeo

**Affiliations:** 1 Technical Textile & Materials R&D Group, Korea Institute of Industrial Technology, 143 Hanggaulro, Sangnok-gu, Ansan-si 15588, Korea; ahndamiro94@kitech.re.kr (D.A.); hjchoi@kitech.re.kr (H.-J.C.); 2 Department of Fiber System Engineering, Dankook University, 152 Jukjeon-ro, Suji-gu, Yongin-si 16890, Korea; hodong@dankook.ac.kr

**Keywords:** polyacrylonitrile, carbon black, wet spinning, conductive fiber, tensile strength

## Abstract

Composites of carbon black (CB) and polymers are attractive for producing conductive fibers. Herein, to achieve improved interactions with polymers, the surface of CB was modified to form 4-aminobenzoyl-functionalized carbon black (ABCB), benzoxazine-functionalized carbon black (BZCB), and Ag-anchored carbon black (Ag-ABCB). The surface-modified CBs were characterized by Fourier transform infrared spectroscopy and thermogravimetric analysis, and X-ray photoelectron spectroscopy was utilized to confirm the presence of Ag in Ag-ABCB. Conductive polyacrylonitrile (PAN) fibers were wet-spun with conductive fillers (CB, ABCB, Ag-ABCB, and BZCB) to investigate the effects of various functional groups on the electrical and mechanical properties. After annealing the conductive PAN fibers, the conductivity and tensile strength greatly increased, whereas the diameter decreased. Notably, the fiber with a BZCB/PAN weight ratio of 12/88 possessed a conductivity of 8.9 × 10^−4^ S/cm, and strength of 110.4 MPa, and thus the highest conductivity and best mechanical properties in the conductive PAN fiber. These results indicate that the annealed BZCB/PAN fibers have potential applications in the manufacturing of antistatic fabrics.

## 1. Introduction

Recently, the convergence of electronic devices and IT technology has advanced the global development of smart textiles with essential core materials made up of conductive fibers. Electrically conductive fibers have various applications depending on the electrical conductivity range; for example, antistatic applications (10^−5^ to 10^0^ S/cm), electric heating (10^−2^ to 10^2^ S/cm), electric wires (>10^3^ S/cm), wearable sensors (10^0^ to 10^1^ S/cm), and stealth applications (10^0^ to 10^2^ S/cm). It is expected that the demand for electrically conductive fibers will continue to increase in the future [1,2,3,4,5].

There are several methods to fabricate conducting fibers. Some of the established methodologies are the formation of a metal layer on the surface of a non-conducting fiber; adding conducting agents such as carbon black (CB), carbon nanotubes, graphene, Cu, or Ag to polymer chains; and spinning metal fibers. Among these methods, the deposition or plating of conductive material on the surface of a nonconductive fiber is advantageous because the fibers produced tend to exhibit high conductivity and good processability. However, there are also a few disadvantages associated with this, such as weak stabilities toward metal oxidation, susceptibility to friction and wear, and the use of large amounts of non-recyclable chemicals during the manufacturing process. On the other hand, the spinning of conductive materials is not competitive in cost and it remains difficult to industrialize owing to problems with corrosion resistance and processability. However, the method of adding conducting agents to polymer chains provides excellent stability and processability, and it has been studied for a long time [6,7,8,9,10,11].

CB is a polycrystalline material in which spherical particles are obtained by the incomplete combustion or pyrolysis of hydrocarbons entangle in grape-like clusters. CB is inexpensive and has high dispersibility and processability. It is commercially available as a rubber reinforcing additive, pigment, conductive additive, etc. [12,13,14]. There are several manufacturing methods for CB/polymer composite fibers, such as solution spinning, melt spinning, electrospinning, and bubbfil spinning. In an earlier study, we reported the fabrication and electrical properties of fibers with functionalized CB added to the PET matrix using melt spinning [15]. However, the melt spinning method has a disadvantage in that the content of the filler in the polymer matrix is limited. Therefore, wet spinning has been commonly used in conductive fiber research because greater amounts of filler can be injected than melt spinning, and dispersion can be achieved in a variety of ways. Besides, it is possible to spin the polymers, which cannot melt, and various studies have been undertaken [16,17,18].

To spin conductive CB/polymer composite fibers, the CB material must reach a percolation threshold while maintaining sufficient dispersibility to pass through the spinning nozzle. Many studies have focused on spinning methods using different types of polymer matrices with CB. However, even when the fibers produced exhibit electrical conductivity, they tend to have poor physical properties because of CB in the fiber agglomerates. Therefore, it is necessary to develop CB materials with high tensile strength and high electrical conductivity by tailoring its interactions with the polymer to produce versatile fibers [19,20,21,22,23].

In this study, we investigated the electrical and mechanical properties of polyacrylonitrile (PAN) fibers prepared using modified CB materials as conductive fillers. The effects of various surface functional groups on CB, including 4-aminobenzoyl groups, benzoxazine groups, and Ag metal, were compared.

## 2. Materials and Methods

**Figure 1:**
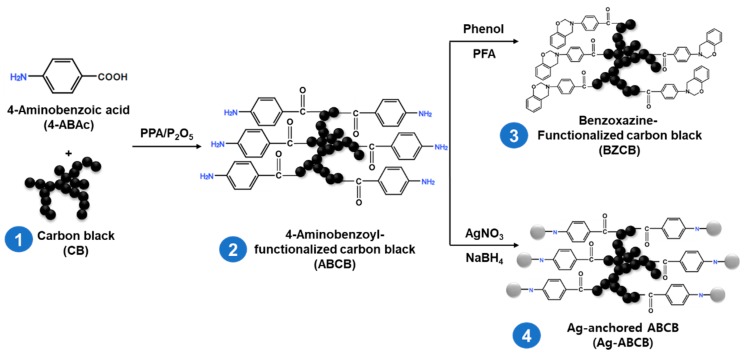
Schematic representation of the carbon black (CB) modification processes.

For our studies, we used polyacrylonitrile (PAN, P-30T, Shaoxing Gimel Advanced Materials Technology Co., Ltd., Shaoxing, China) as the polymer matrix and *N*,*N*-dimethylformamide (DMF, 99%, Daejung Chemical Co., Ltd., Shiheung, Korea) as the solvent. CB (Ketjenblack EC 600JD, AkzoNobel, Amsterdam, Netherlands) was modified using the methods outlined in Figure 1. The 4-aminobenzoic acid (≥99%, 4-ABAc), sodium borohydride (>98%, NaBH_4_), phenol (99.0–100.5%), *para*-formaldehyde (95%, PFA), and ethanol (EtOH) were obtained from Sigma Aldrich Chemical Inc. (St. Luis, MO, USA), and used without any further treatment. The polyphosphoric acid (83% P_2_O_5_ assay, PPA), phosphorus pentoxide (98%, P_2_O_5_), 1,4-dioxane (99%), and silver nitrate (AgNO_3_) were purchased from Daejung Chemical Co., Ltd.

### 2.1. CB Modification

#### 2.1.1. 4-Aminobenzoyl-Functionalized Carbon Black (ABCB)

We adopted the synthesis method reported by Lee et al. [24] and Choi et al. [15]. In brief, a mixture of 4-ABAc (3.65 mmol), CB (20 g), PPA (1000.0 g), and P_2_O_5_ (250.0 g) was placed in a 2 L resin flask equipped with a high-torque mechanical stirrer and a nitrogen inlet and outlet. The reaction was carried out stepwise first at 80 °C for 6 h; then, at 100 °C for 10 h; and finally, at 130 °C for 72 h under nitrogen atmosphere. The mixture was poured into distilled water, and the resulting powdery product was collected by suction filtration and Soxhlet-extracted with water for 3 days to completely remove the residual reaction medium; and once again, Soxhlet-extracted with ethanol for 1 day to remove the unreacted 4-ABAc. Finally, the obtained mass was freeze-dried under reduced pressure (0.05 mmHg) for 3 days.

#### 2.1.2. Benzoxazine-Functionalized Carbon Black (BZCB)

BZCB was synthesized following the method of Andreu et al. [25]. ABCB (5 g), phenol (5 g), and PFA (3.5 g) in a molar ratio of 1:2:4 were refluxed in 1,4-dioxane (650 mL) under magnetic stirring for 5 days. The resultant powdery product was collected by suction filtration and then Soxhlet-extracted with water for 3 days to completely remove residual reaction medium and unreacted monomers. Finally, the sample was freeze-dried under reduced pressure (0.05 mmHg) for 3 days.

#### 2.1.3. Ag-Anchored ABCB (Ag-ABCB)

To prepare Ag-ABCB, ABCB (10 g) was rapidly dispersed in AgNO_3_ solution (0.5 mmol in EtOH). Then, NaBH_4_ (0.1 mmol in EtOH) was slowly added drop wise, resulting in the attachment of Ag to the ABCB surface (Ag-ABCB). Finally, the resulting product was washed several times with distilled water and the sample was freeze-dried under reduced pressure (0.05 mmHg) for 3 days.

### 2.2. Preparation of Fibers

**Figure 2:**
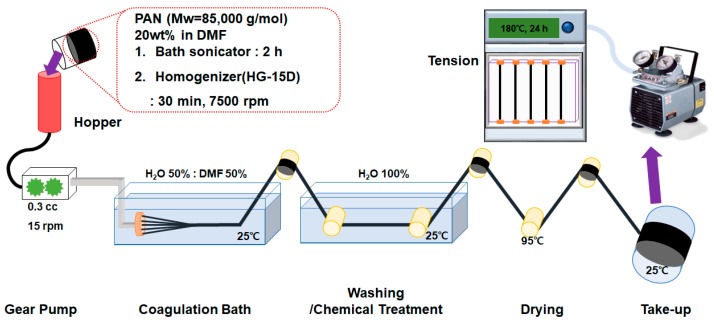
Schematic of the manufacturing processes for conductive fibers.

The solutions for spinning PAN were prepared by dissolving the 20 wt % of the polymer in DMF. Then, CB (10 wt %), ABCB (12 wt %), BZCB (12 wt %), or Ag-ABCB (20 wt %) was dispersed in the polymer solution using a homogenizer (HG-15D, Daihan Scientific Co., Ltd., Wonju, Korea) at 7500 rpm for 30 min at room temperature. The dispersed solution was allowed to stand for 1 day at room temperature for degassing and then directly used for wet-spinning. The spun fibers were heat-treated in a vacuum oven at 180 °C for 24 h under constant tension. Figure 2 shows the schematic of the fiber manufacture processes and the conditions applied for each process.

### 2.3. Characterization

Various spectroscopic techniques were employed to analyze the surface chemical compositions of the modified CBs, which includes Fourier transform infrared (FT-IR) spectroscopy (Spectrum Two, Perkin-Elmer, Waltham, MA, USA) and X-ray photoelectron spectroscopy (XPS, K-alpha, ThermoFisher, Waltham, MA, USA). For the FT-IR measurements, the CBs were pelletized with potassium bromide (KBr) as the diluent and the spectra were collected in the scan range of 500–4000 cm^−1^. The XPS analysis was conducted in the range of 0–3000 eV. Thermogravimetric analysis (TGA) was performed using a thermogravimetric analyzer (Q500, TA Instruments, New Castle, DE, USA) from 25 to 800 °C at a heating rate of 20 °C/min under airflow and nitrogen atmosphere. Field emission-scanning electron microscopy (FE-SEM; SU8000, Hitachi Ltd., Tokyo, Japan) was used to confirm the morphology of the synthesized conductive filler and degree of dispersion in the PAN matrix. All samples were cut after pre-treatment with liquid nitrogen. Energy dispersive X-ray spectroscopy (EDX) was measured by Hitachi S-4700.

Electrical conductivity measurements were conducted using various instruments, depending on the magnitude of the conductivity. Fiber-type samples were manufactured by stacking 25 filaments on a glass slide and then covering the filaments with silver paste at intervals of 1 cm. For resistances greater than 10^−5^ Ω/cm, measurements were performed using a Keithley 6517B high-resistance meter (Keithley Instruments Inc., Cleveland, OH, USA). For resistances less than 10^−5^ Ω/cm, measurements were performed using a 3280-20F Clamp On HiTester (Hioki, Japan). The measurements were based on the ASTM-D-257 standard test methods, which is a 2-point method. The resistance, *ρ*, was calculated using Equation (1), where *R*, *A*, *l*, and *n* are the measured resistance between the electrodes, the cross-sectional area of the fiber, the distance between the electrodes, and the number of fibers, respectively [26,27]. The electrical conductivity of the fiber, *σ*, was calculated from the resistance using Equation (2).

(1) $$\rho \left(\Omega \cdot \mathrm{cm}\right)=R\left(\Omega \right)\times \;\frac{A\;\left(cm^{2}\right)}{l\;\left(cm\right)}\;\times \;\frac{1}{n}.$$

(2) $$\sigma \left(S/\mathrm{cm}\right)=\frac{1}{\rho \left(\Omega \cdot \mathrm{cm}\right)}.$$

The Single fiber testing was performed using the Favimat (Textechno, Mönchengladbach, Germany), which measured tensile strength of PAN, CB10/PAN, ABCB12/PAN, BZCB12/PAN, and Ag-ABCB20/PAN fibers. A gauge length of 20 mm and cross-head speed of 20 mm/min were used. The tenacity was the average value of 10 repeated measurements.

## 3. Results and Discussion

### 3.1. CB Functionalization

Figure 1 shows the CB modification scheme. ABCB was prepared using ABAC to functionalize CB via a Friedel–Crafts acylation reaction. Friedel–Crafts acylation in the PPA/P_2_O_5_ medium is milder and less corrosive than other functionalization systems. Thus, this reaction does not damage the graphitic framework, allowing the preservation of the intrinsic properties of the graphitic materials [28].

BZCB was synthesized by a Mannich reaction of phenol, para-formaldehyde, and the primary amines of the aminobenzoyl group of ABCB. As BZCB can undergo ring-opening processes in the absence of an initiator or strong and toxic acid catalysts, this material can be easily bonded to PAN via heat treatment [29,30].

It is known that metal–carbon composites are advantageous for the preparation of high-conductivity composite fibers [15]. Thus, to fabricate a metal–carbon composite (Ag-ABCB), Ag was attached to the surface of the ABCB using AgNO_3_ as a silver precursor and NaBH_4_ as the reducing agent.

**Figure 3:**
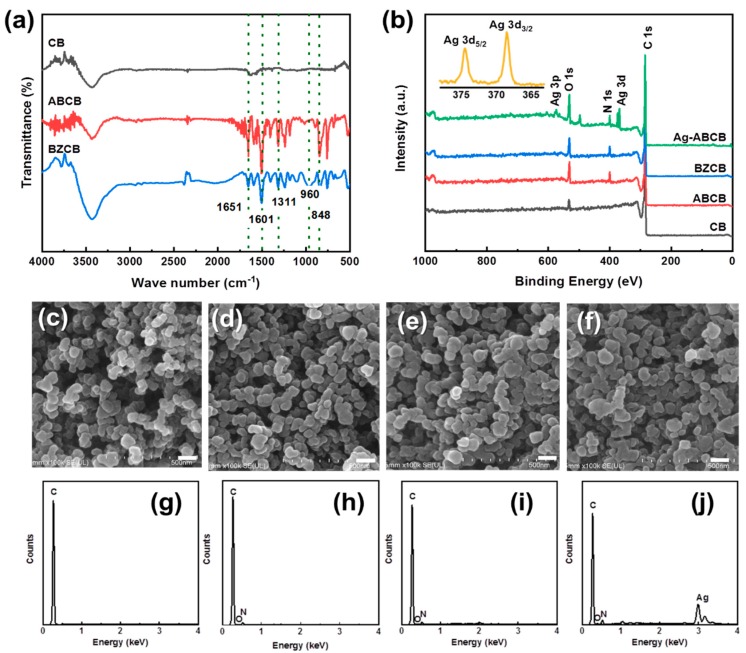
(**a**) FT-IR and (**b**) XPS spectra of CB, ABCB, and BZCB; SEM images of (**c**) CB, (**d**) ABCB, (**e**) BZCB, and (**f**) Ag-ABCB. Scale bar is 100 nm. EDX analysis of (**g**) CB, (**h**) ABCB, (**i**) BZCB, and (**j**) Ag-ABCB.

The FT-IR spectra of pristine CB and the modified CBs are presented in Figure 3a. There were no significant functional groups detected on pristine CB. However, after modification, the peaks corresponding to various functional groups were observed. For ABCB and BZCB, an apparent keto-carbonyl band was observed near 1651 cm^−1^, which clearly shows the covalent attachments of the organic moieties to the surface of CB. Other characteristic peaks confirmed the presence of primary amines (N–H bending vibration at 1603 cm^−1^) and aromatic amines (C–N stretching band at 1311 cm^−1^ and N–H out-of-plane bending vibration at 848 cm^−1^). Additionally, the peaks observed for BZCB at 910–960 cm^−1^ were not observed for ABCB, confirming the presence of a benzoxazine ring in BZCB [24]. These results indicated that the CB was well modified by the 4-ABAc and benzoxazine group to produce ABCB and BZCB.

The XPS spectra of CB before and after modification are shown in Figure 3b. The CB sample exhibits only two peaks corresponding to C 1s and O 1s at binding energies of 297.5 and 545 eV, respectively. For ABCB and BZCB, the N 1s peak was also apparent (at 403 and 410 eV, respectively), and the intensity of the O 1s peak increased. These results confirm that the CB surface was successfully modified. Additionally, XPS was used to characterize Ag in Ag-ABCB (Figure 3b). The existence of two Ag 3d peaks for Ag-ABCB at binding energies of 368.3 and 374.3 eV with a difference of 6.0 eV confirmed the formation of metallic silver. The standard Ag 3d_5/2_ and Ag 3d_3/2_ binding energies of pure silver are 368.1 and 374.1 eV, respectively, and the shift of the Ag 3d peaks of Ag-ABCB to lower binding energies may be due to the electron transfer between the Ag nanoparticles and CB [31].

SEM images show the morphologies of CB, ABCB, BZCB, and Ag-ABCB. Agglomerated particle sizes of approximately 60–70, 70–80, and 70–80 nm were determined for the CB, ABCB, and BZCB respectively (Figure 3c–e). The particle size gradually increased in the order CB < ABCB < BZCB because of 4-ABAc and benzoxazine functionalization. The Figure 3g–j shows the EDX of conductive fillers. The pristine CB showed a C peak and after the functionalization 4-ABAc; O and N peaks newly emerged. The benzoxazine functionalized showed C, O, and N peaks from CB and the benzoxazine group. In the Ag, anchored ABCB and Ag peaks appeared. These results strongly indicate the functionalization of the 4-aminobenzoyl group and benzoxazine group, and attachment of Ag particle on the ABCB. Besides, the bright and white parts indicated by the arrows showed that the partially aggregated silver nanoparticles were well loaded to the ABCB surface (Figure 3f). The inset in Figure 3f shows Ag peaks from Ag-ABCB nanocomposites, visually showing the effective attachment of the Ag nanoparticles to the ABCB’s surface.

### 3.2. Thermal Properties of Modified CB

**Figure 4:**
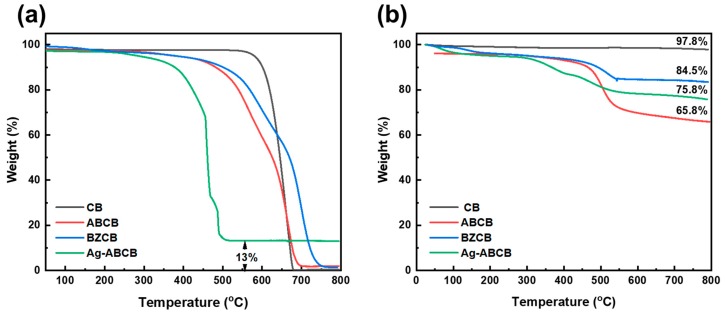
TGA thermograms of modified CBs in (**a**) air and (**b**) N_2_.

The TGA thermograms of CB and the modified CBs are shown in Figure 4. Thermal decomposition of the materials upon heating was examined under air (Figure 4a) and nitrogen atmospheres (Figure 4b). The thermo-oxidative stabilities of the samples were demonstrated by the thermograms in air, as shown in Figure 4a. Pristine CB showed no weight change until approximately 620 °C, and then the weight decreased rapidly. However, in the cases of ABCB and BZCB, primary thermal decomposition occurred at 570 and 605 °C with secondary thermal decomposition occurring at 670 and 698 °C, respectively. The difference between the pyrolysis behavior of pristine CB and the modified CBs in the air is likely due to the organic moieties covalently attached to the surfaces of the modified CBs, which undergo thermal decomposition at lower temperatures, observed as the primary thermal decomposition process. Analogous to transformations observed during carbonization, during BZCB pyrolysis, the benzoxazine groups form aromatic rings that can react with defects on the CB surface. This process inhibits thermal decomposition during high-temperature charring, and thus improves the thermal stability of BZCB compared with those of the other modified CBs [32]. The char yield of Ag-ABCB at 800 °C in air was approximately 13 wt %, whereas those of the other samples showed values close to 0 wt %. The residual amount of Ag-ABCB at 800 °C should be related to the Ag particles, and this amount was 13 wt %. This is a strong indication that the Ag particles were successfully attached to the surface of the ABCB and that the residual char yield at 800 °C was due to the amount of Ag particle loaded [15].

As shown in the TGA thermograms obtained under a nitrogen atmosphere (Figure 4b), CB displayed linear weight loss behavior, with a char amount of 97.8 wt % at 800 °C. In contrast, owing to their organic functional groups, ABCB and BZCB exhibited weight losses in nitrogen starting at 450–500 °C, with char amounts at 800 °C of 65.8 and 84.5 wt %, respectively. The difference in the amounts of char for these samples supports the speculation that BZCB may fix the defects on the surface of CB and prevent CB thermal decomposition during charring. Additionally, the char amount of Ag-ABCB at 800 °C (75.8 wt %) was approximately 10 wt % higher than that of ABCB. This difference is similar to that observed in the air atmosphere, suggesting that it corresponds to the amount of Ag particles in Ag-ABCB.

### 3.3. Electrical Properties of Modified CB/PAN Fibers

**Figure 5:**
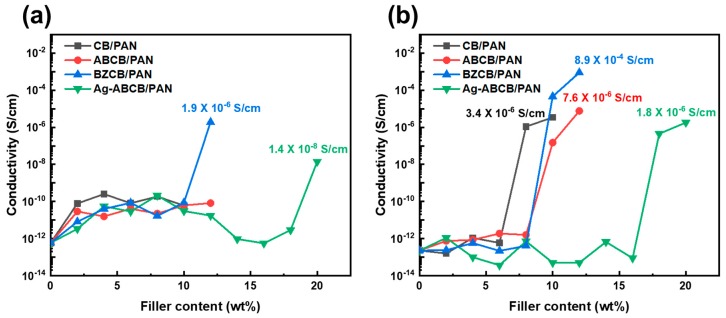
Electrical conductivities of PAN fibers with different contents of CB and modified CBs (**a**) as-spun and (**b**) annealed fiber.

The electrical conductivities of the modified CB/PAN fibers are shown in Figure 5. The conductivity values were calculated using Equation (2) using the resistances of the fibers measured as-spun (Figure 5a) and annealed fiber (Figure 5b). The sample names indicate the type and content of CB material in the fiber; for example, the fiber with 10 wt % pristine CB as filler is denoted by CB10/PAN. As shown in Figure 5a, the CB/PAN and ABCB/PAN fibers show no change in electrical conductivity, even at the highest filler content. However, BZCB12/PAN and Ag-ABCB20/PAN exhibited significant electrical conductivity values of 1.9 × 10^−6^ and 1.4 × 10^−8^ S/cm, respectively. Even at the same filler content, the electrical conductivity of BZCB12/PAN is higher than that of ABCB12/PAN. Various studies have reported the use of benzoxazine resins as a noncovalent dispersant [32]. Thus, modification of the CB surface with benzoxazine groups may weaken the van der Waals forces between CB particles, which can improve dispersibility and enhance electrical conductivity. Moreover, the Ag-ABCB/PAN fibers exhibit enhanced electrical conductivity because the Ag particles have a higher electrical conductivity than CB [33].

As shown in Figure 5b, the annealed CB10/PAN, ABCB12/PAN, BZCB12/PAN, and Ag-ABCB20/PAN fibers exhibit maximum electrical conductivity values of 3.4 × 10^−6^, 7.6 × 10^−6^, 8.9 × 10^−4^, and 1.8 × 10^−6^ S/cm, respectively. After annealing, all the fibers exhibited a percolation threshold; i.e., the point at which the electrical conductivity sharply increased. It is known that annealing causes phase coarsening to occur, which will drive the CB nanoparticles to self-assemble into a nanoparticle network, making the CB/polymer composites conductive. The electrical conductivities of the BZCB/PAN and Ag-ABCB/PAN fibers, which exhibited electrical conductivity as-spun fiber, did not increase significantly. The formation of the CB conductive network results in strong interactions between the CB particles, and thus, the morphologies of the CB/composites are fixed by this network structure. However, this effect is less prominent when it occurs at a CB content above the percolation threshold [34,35].

### 3.4. Effect of Annealing on Fiber Diameter

**Figure 6:**
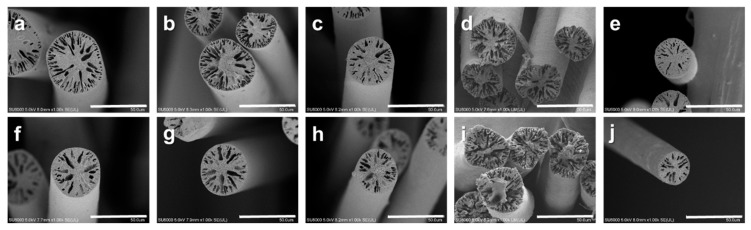
SEM images of as-spun and annealed fibers. As-spun fibers: (**a**) PANB, (**b**) CB10/PAN, (**c**) ABCB12/PAN, (**d**) BZCB12/PAN, and (**e**)Ag-ABCB20/PAN. And annealed fiber: (**f**) PANB, (**g**) CB10/PAN, (**h**) ABCB12/PAN, (**i**) BZCB12/PAN, and (**j**) Ag-ABCB20/PAN.

**Figure 7:**
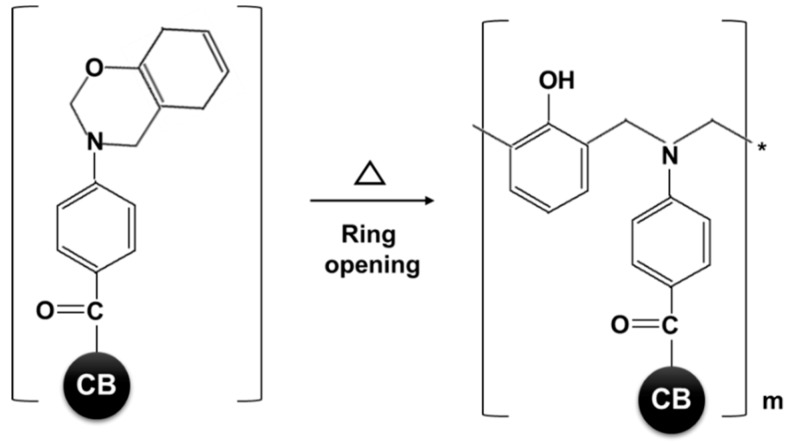
Schematics of ring-opening reaction of the benzoxazine groups in BZCB during annealing.

Figure 6 and Table 1 compares the mean diameters of the as-spun and annealed PAN fibers with various CB fillers, as determined from surface SEM images. The chosen content for each filler corresponded to the fibers that exhibited the most pronounced characteristics. The diameters of all the fibers decreased after the annealing process. Especially, the diameters of the as-spun fiber were PAN 53.9 µm and Ag-ABCB20/PAN 43.6 µm, but the diameters of the annealed fibers largely decreased, with values of PAN 41.4 µm and Ag-ABCB20/PAN 34.1 µm. In general, when fibers are annealed, the diameter decreases owing to heat shrinkage [36]. As shown in Figure 6, the diameter reduction ratios of the fibers reinforced with fillers were lower than that of the as-spun PAN fiber. Even though the filler content was the same, the diameter reduction ratio of BZCB12/PAN was lower than that of ABCB12/PAN. This was due to the evolution of annealing by-products of different volatilities and pore generation through the polymeric matrix. As shown in Figure 7, when BZCB is heated, the benzoxazine group can undergo a ring-opening reaction, allowing the formation of covalent bonds or hydrogen bonds with the nitrile groups of PAN. It is reasonable to expect the formation of such bonds to alter the diameter owing to heat shrinkage of the fiber; therefore, the diameter reduction ratio of BZCB12/PAN decreased [30,31].

### 3.5. Mechanical Properties of Modified CB/PAN Fibers

**Figure 8:**
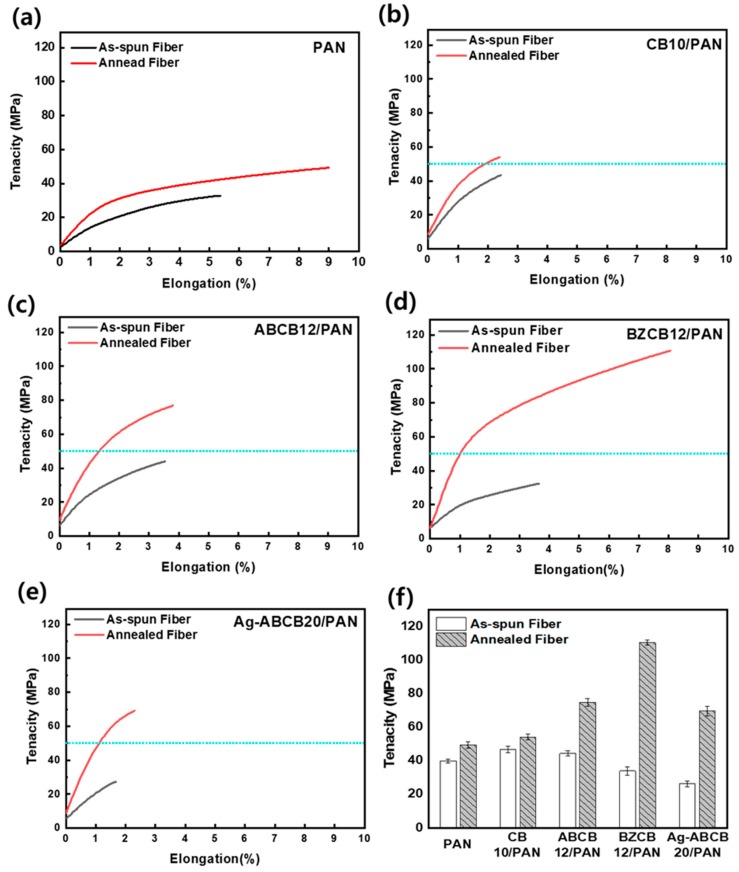
Tensile properties of as-spun PAN fiber and annealed PAN fibers at 180 °C: (**a**) PAN, (**b**) CB10/PAN, (**c**) ABCB12/PAN, (**d**) BZCB12/PAN, and (**e**) Ag-ABCB20/PAN; (**f**) tenacity of the as-spun and annealed fibers. The sky blue line shows annealed PAN fiber with 49.3 MPa tenacity.

The uniaxial stress–strain curves of PAN fiber changed with annealing and varying CBs surface are shown in Figure 8. The PAN fiber and the composite fibers containing the carbon black additives increased the tenacity of the annealed fibers compared to the as-spun fibers. In particular, the tenacity (110.4 MPa) of the BZCB12/PAN annealed fiber was increased almost four times higher than that (33.9 MPa) of the as-spun fiber. In general, it is known that the tensile strengths of fibers increase after annealing owing to the decrease in the fiber diameter caused by heat shrinkage as well as a decrease in the defects in the fiber introduced during the wet-spinning process. These phenomena explain the increase in the strength from 32.9 MPa to 49.3 MPa of the annealed PAN fiber [37].

The mechanical property changes after the annealing process are discussed in the sense of the difference in the chemistry of the CBs surface. The general tendency is that the stress level increases by the addition of CBs, which play the role of reinforcement. The same result was obtained for all the fibers with CBs and was stronger than the PAN fiber. The effects of reinforcement, such as elongation, modulus, and strength of the organic modified CBs, were higher than fiber with inorganic modified CBs. Especially, the reinforcement effect of CBs with organic functional groups was a noticeable elongation of the fiber. It means that the tensile properties of the fiber might have a positive gain from the CBs surface if the interface between the CBs and fiber was secured. In particular, the tensile properties of BZCB12/PAN were the highest after annealing, which, as mentioned above, may be explained by the formation of bonds between the PAN fiber and the filler.

## 4. Conclusions

In the present study, CB was modified with 4-aminobenzoyl groups, benzoxazine groups, or Ag nanoparticles. FT-IR and XPS analyses confirmed the presence of organic functional groups and Ag on the modified CBs. Furthermore, TGA revealed that BZCB showed high thermal stability and indirectly confirmed the presence of Ag particles in Ag-ABCB. Using CB and the modified CBs as fillers, CB/PAN and modified CB/PAN composite fibers were successfully produced. The electrical and mechanical properties of the composite fibers depend on the surface functional groups on CB. Both the electrical conductivities and tensile strengths of the PAN fibers increased after annealing. After annealing, BZCB12/PAN had the highest electrical conductivity and tensile strength (8.9 × 10^−4^ S/cm and 110.4 MPa, respectively). Furthermore, the fiber diameters decreased upon annealing, and the diameter reduction ratio was lowest for BZCB12/PAN. Owing to the benzoxazine groups in BZCB, the annealing process led to bond formation between PAN and BZCB in the BZCB/PAN composite fiber. As a result, BZCB12/PAN exhibited the best properties among the manufactured conductive fibers.

## Figures and Tables

**Table 1:** Diameters of as-spun and annealed fibers.

Sample	Diameter (µm)				
	PAN	CB10/PAN	ABCB12/PAN	BZCB12/PAN	Ag-ABCB20/PAN
As-spun Fiber	53.9 ± 1.3	48.5 ± 1.3	43.6 ± 1.0	39.9 ± 0.5	43.6 ± 0.9
Annealed Fiber	41.4 ± 2.3	41.9 ± 0.4	36.7 ± 1.5	36.2 ± 0.6	34.1 ± 0.5
